# Spanish Validation of the Shorter Version of the Workplace Incivility Scale: An Employment Status Invariant Measure

**DOI:** 10.3389/fpsyg.2018.00959

**Published:** 2018-06-13

**Authors:** Donatella Di Marco, Inés Martínez-Corts, Alicia Arenas, Nuria Gamero

**Affiliations:** ^1^Business Research Unit (BRU-IUL), Instituto Universitário de Lisboa (ISCTE-IUL), Lisbon, Portugal; ^2^Department of Social Psychology, University of Seville, Seville, Spain

**Keywords:** workplace incivility scale, Spanish validation, invariant measure, shorter version, employment status, employees’ well-being

## Abstract

Workplace Incivility (WI) occurs worldwide and has negative consequences on individuals and organizations. Valid and comprehensive instruments have been used, specifically in English speaking countries, to measure such adverse process at work, but it is not available a validated instrument for research carried out in Spanish speaking countries. In this study we aim to test the psychometric properties of the Matthews and Ritter’s four-item Workplace Incivility Scale (2016) with Spanish workers (*N* = 407) from different sectors. Participants’ mean age was 38.73 (*SD* = 10.45) years old and the percentage of female employees was 59.2%. Confirmatory factor analysis using AMOS 19.0 was carried out, presenting a good fit. The internal consistency, convergent and concurrent validity of the scale were examined. Results show good scale reliability and expected high correlations with social undermining. Moreover, the scale related to propensity to leave a job, job satisfaction, and psychological well-being in the expected way. After configural invariance across groups was established, testing for metric invariance and scalar invariance was performed. Considering Δχ^2^ and ΔCFI tests for two nested models, the 4-item scale was invariant when the employment status is considered (permanent vs. temporal, full-time vs. part-time, and supervisor vs. non-supervisors). Overall, our findings showed good psychometric properties of the shorter version of the WIS in Spain. Theoretical and practical implications of this study are discussed.

## Introduction

### Workplace Incivility

Workplaces are spaces characterized by rules defined as appropriate in a specific society. Sometimes such rules might be broken and people might carry out behaviors considered as deviant at that moment and in that context (Pawar, 2013), affecting negatively employees’ well-being and satisfaction. In the last few years, several researchers have focused their attention on deviant behaviors at work and many of them have been identified (e.g., Hershcovis, 2011): social undermining (Duffy et al., 2002), bullying (Hoel et al., 1999; Einarsen, 2000), ostracism (Williams, 2007), or abusive supervision (Tepper, 2000) are only some of them. Quite recently, workplace incivility (WI), defined as “low-intensity deviant behavior with ambiguous intent to harm the target, in violation of workplace norms for mutual respect” (Andersson and Pearson, 1999, p. 457), has captured the attention of many researchers. WI is characterized by the ambiguity about the real intention of the perpetrator. In fact, uncivil acts might be the result of an intentional mistreatment; but it might also be the consequence of a lack of nicety (Pearson et al., 2000) without any goal of harming the other part. Moreover, following the original definition, WI might pass as unseen, given the low-intensity of acts that enter in its domain. In the original works of (Andersson and Pearson, 1999; Pearson et al., 2001) and in the following research on this issue (e.g., Cortina et al., 2001, 2013) behaviors of different nature where identified as WI. Shouting against a coworker, isolating him or her, devaluating a colleague’s job, making jokes at others’ expense: all these behaviors enter into the realm of what is defined as WI. The subtlety of such acts makes difficult to define them as mistreatments and can be attributed to a rude environment (Leiter, 2013). Various researchers (e.g., Hershcovis, 2011) have pointed out the growth and, often, the overlapping between constructs related to negative acts at work. One of them is social undermining (Duffy et al., 2002) which refers to those behaviors whose intent is undermining victims’ work relational ties and reputation. Although related concepts, they can be differentiated by the intent to harm which is clearer in social undermining and ambiguous in case of incivility.

Although uncivil acts are not blatantly aggressive and victims often are not aware of the perpetrator’s intent to harm, they might be the cause of several prejudicial consequences for people’s well-being and organizations (Di Marco et al., 2015; Garrosa et al., 2015; Kabat-Farr et al., 2016; Schilpzand et al., 2016). Such consequences depend on the intensity of the act and its frequency (Hershcovis, 2011; Leiter, 2013). Incivility is an organizational stressor (Griffin, 2010) and being victim of uncivil behaviors produces negative outcomes. Previous studies demonstrated that WI affects physical and psychological well-being, diminishes job satisfaction and increases the desire of revenge (Andersson and Pearson, 1999; Cortina et al., 2001; Pearson et al., 2001; Diaz et al., 2011; Moreno-Jiménez et al., 2012; Kabat-Farr et al., 2016); it also reduces job performance, increases the intention to leave and organizational costs (Griffin, 2010; Porath and Pearson, 2010; Taylor et al., 2012; Schilpzand et al., 2016; Mao et al., 2017). Moreover, recent research has demonstrated that being victim of WI can lead to counterproductive work behaviors (voluntary actions which are prejudicial for the organization or their members) (Sakurai and Jex, 2012; Welbourne and Sariol, 2017), increasing the cost of incivility for the organization. People who perceive WI spend more time thinking on uncivil acts experienced, are less satisfied and the probability to quit the organization is higher (Cortina et al., 2001).

The cost of WI is paid by victims and organizations, but also by bystanders and all those people who enter into the uncivil spiral (Montgomery et al., 2004; Porath and Erez, 2009). In fact, not paying attention to WI might involve the escalation of conflict. Workers who are victims of uncivil acts might ignore such treatment or might desire to retaliate with the original perpetrator or with another person. Indeed WI might be contagious (Andersson and Pearson, 1999) and a spiral of incivility might rise, involving many people within the organization. All the organizational actors might be implicated. Past studies found that supervisors, coworkers and clients might perpetrate incivility (Schilpzand et al., 2016). Specially, people at the top of the hierarchy might use more often incivility as a mean to exercise power or to push productivity (Porath and Pearson, 2010; Kabat-Farr et al., 2017).

### Workplace Incivility Measure

Rudeness and uncivil behaviors are largely widespread at the workplace and many workers confirmed they have been victims of them at least once in their working life (Cortina et al., 2001; Pearson and Porath, 2013). In order to measure the impact of this phenomenon, numerous qualitative (Andersson and Pearson, 1999; Pearson et al., 2001; Di Marco et al., 2015) and quantitative (Welbourne et al., 2015) studies have been carried out. Several scales have been developed with the goal of creating a comprehensive instrument in order to measure quantitatively and objectively uncivil acts at the workplace (e.g., Cortina et al., 2001, 2013; Martin and Hine, 2005). However, “WI is not an objective phenomenon; it reflects people’s interpretation of how actions make them feel” (Porath and Pearson, 2010, p. 64) and several factors might play a role in the process of interpretation of behaviors considered uncivil (e.g., hierarchical status) (Schilpzand et al., 2016).

Although different measures have been developed (e.g., Cortina et al., 2001, 2013; Martin and Hine, 2005; Wilson and Holmvall, 2013), Cortina and colleagues’ Workplace Incivility Scale (WIS), in the original (Cortina et al., 2001) and revised version (Cortina et al., 2013) is the most known and applied instrument to date. The WIS is a unidimensional scale that brings together 7 (Cortina et al., 2001) or 12 items (Cortina et al., 2013) which refer to several uncivil behaviors experienced in a variable recall window: “the past 5 years” in the former version, and “the last year” in the latter. In fact, researchers were conscious that results can be affected significantly by the length of the recall window (Blau and Andersson, 2005; Matthews and Ritter, 2016), due to distractions that a longer recall window might create (Matthews and Ritter, 2016).

There have been several attempts to reduce or adapt the WIS to different research goals (e.g., Blau and Andersson, 2005; Matthews and Ritter, 2016). Recently, Matthews and Ritter (2016) elaborated a shorter (four-item scale) WIS based on the second version of Cortina et al.’s (2013) WIS. Applying a shorter instrument is important in order to improve the clarity of the construct that researcher would measure; it enhances the probability that respondents complete the questionnaire, even more when several scales are applied at the same time (Fisher et al., 2015; Balducci et al., 2017). Reduced scales are necessary when researchers carry out longitudinal studies and, also, when responses are given by computer, due to the largely use of electronic surveys (Balducci et al., 2017).

The four item-reduced version of the WIS (Matthews and Ritter, 2016) was the result of a complex analysis which first had the goal to explore the judgmental qualities of the scale, understanding which items of the 12-items WIS version (Cortina et al., 2013) respected the initial conceptualization that Andersson and Pearson (1999) gave to the construct. In other terms, researchers explored if both the distinguishing features of the original construct -the ambiguous intent to harm and the low intensity of acts- were reflected by the WIS. Then convergent and divergent validity were analyzed and the four items of the reduced version were identified. The reduced WIS showed good psychometric properties with a Cronbach’s α = 0.79 and factor loadings ranged from 0.57 to 0.77. The reduced scale correlated 0.93 with the 12-items scale and both explained similar levels of variance in the dependent variables analyzed (e.g., civility norms, interpersonal deviance, organizational deviance, etc.).

The goal of this research is to validate a Spanish version of the WI reduced scale elaborated by Matthews and Ritter (2016). The recent increment of workers’ demands and the escalation of work insecurity due to the financial crises have involved several countries. For instance, in Spain, only 59% of workers perceive job security (Eurofound, 2017). According the 6th Eurofound Working Conditions Survey, 16% of European workers perceived adverse social behavior at work and 16% have not been treated fairly in their workplace. In Spain the prevalence of these issues is 10 and 21%, respectively (Eurofound, 2017). Thus, this trend might have reinforced negative acts such as WI which might be instigated by a demanding workplace (Schilpzand et al., 2016). The few studies on WI in Spain showed that uncivil behaviors generate workers less satisfied, who carried out more counterproductive behaviors (Diaz et al., 2011); the emotional exhaustion increases (Garrosa et al., 2015) as well as the intention to leave the organization (Moreno-Jiménez et al., 2012). Although research carried out in Spain to date does not show high levels of incivility, they highlight its prejudicial effects at individual and organizational level. In order to recognize and evaluate the impact of WI, it is necessary to analyze the psychometric properties of the WIS, making available a valid and reduced measure of such construct for those studies carried out in Spain. In the present study we analyze, firstly, the items reliability and factor loading of the Spanish reduced WIS; secondly, we analyze the convergent validity of the reduced WIS with another related concept, such as social undermining; and the concurrent validity with several outcomes propensity to leave, job satisfaction, and psychological well-being. Finally, as Matthews and Ritter (2016) highlighted in their work, it is necessary to obtain an invariant scale based on the characteristics of the employment type. While their measure results to be invariant when applied to full-time versus part-time workers and to men and women, they did not consider other features of the employment, such as the type of contract (permanent vs. temporal contract) and the position occupied in the hierarchical scale (supervisors vs. non-supervisors). In fact, people who are in a temporary contract might be more likely victims of WI, because their transit within the organization is perceived as limited and perpetrators might care less about consequences derived from negative behaviors against this group of workers (Salminen and Saloniemi, 2010). The same process might arise when we are in front of people with different level of formal power. As research has underlined previously (e.g., Schilpzand et al., 2016), subordinates experience WI more than superiors do. Consequently, the goal to validate an invariant measure will be to find out those behaviors that are uncivil beyond the employment status.

## Materials and Methods

### Participants

Participants were 407 employees from a broad range of professional backgrounds, including trade (28%), education (25.3%), insurance (10.8%), health and welfare (8.8%), catering (6.4%), industry (4.7%), culture and leisure (4.2%), construction (3.2%), transport (1.7%), business service (1.7%), agricultural sector (1.2%), government (1.2%), communication (1%), and others (2.8%). The percentage of female employees was 59.2%. Regarding their highest education, 58.5% had a university degree, 30% had completed high school or vocational education, 10.1% had completed elementary studies, and 1.5% had not completed any formal education. Respondents’ mean age was 38.73 (*SD* = 10.45) years old. Most respondent held a fulltime employed (70.3%) and no managerial position (57.5%). About 72% held permanent contract, 18.9% held temporal contract and 2.2% were freelance. Participants worked an average of 26.54 (*SD* = 14.37) hours per week. Their average seniority at the current company was about 12 years (*SD* = 9.87).

### Measures

#### Workplace Incivility Scale

Participants answered the short version of WI by Matthews and Ritter (2016), which comprises 4 items (see **Appendix A**). Respondents had to think about if in the last year they have been victims of uncivil behaviors perpetrated by a coworker or supervisor (e.g., “Made jokes at your expense”). Respondents were scored on a 5-point Likert scale ranging from 1 (never) to 5 (many times). Cronbach’s alpha was 0.75.

#### Social Undermining Scale

Duffy et al. (2002) 26-item measure was used. Using a 6-point Likert scale ranging from 1 (never) to 6 (everyday) participants were asked to think about the frequency their supervisors and coworkers show the listed behaviors (e.g., “Hurt your feelings”). Cronbach’s alpha was 0.96.

#### Propensity to Leave a Job Scale

González-Romá et al. (1992) 3-item measure was used. Using a 5-point Likert scale ranging from 1 (absolutely disagree) to 5 (absolutely agree) participants were asked to think about their workplace and reflect on whether they would change to another job in the same organization (e.g., “I would feel better if I occupied the same position (with the same work conditions) in another department or section at my organization”). Cronbach’s alpha was 0.89.

#### Job Satisfaction Scale

The Minnesota Satisfaction Scale (Weiss et al., 1967) 5-item measure of extrinsically and general satisfaction was used. Using a 5-point Likert scale ranging from 1 (unsatisfied) to 5 (very satisfied) participants were asked to think how satisfied they are with different aspect of their job (e.g., paid, security, coworkers, supervisors) and with the job in general (e.g., “In general, how satisfied are you with your actual job?”). Cronbach’s alpha was 0.74.

#### Psychological Well-Being Scale

The 12-item General Health Questionnaire Goldberg’s (1972), adapted by González-Romá et al. (1991) was used. The scale is measured in a 4-point response format ranging from 1 (rarely) to 4 (frequently). Participants were asked to describe how they feel actually (e.g., “Have you recently felt constantly under strain?”). Cronbach’s alpha was 0.76.

### Translation and Adaptation Procedures

According to the International Test Commission guidelines (Hambleton et al., 2005; International Test Commission [ITC], 2005), four expert researchers first translated the WIS into Spanish trying to preserve the original meaning of items. Second, the translated items were discussed and the four experts got a consensus about the content of the items. In general, item translation did not generate debate and consensus was reached easily. Third, a back-translation was conducted by an English speaking translator and the equivalence between the two versions was assured. The final Spanish version of the scale is shown in “**Appendix A**”.

### Data Collection Procedure

Employees from various organizations in Spain were recruited using an incidental sampling scheme. They voluntary fulfilled a cross-sectional survey in approximately 15 min. Participants were informed about the anonymity and confidentiality of the survey.

### Analyses

Confirmatory factor analysis (CFA) was conducted using AMOS 19.0 and all other statistical procedures were computed with SPSS 24.0. Since there was evidence of data’s deviation of normal distribution and the sample is moderated we computed analysis with Asymptotic Distribution-Free (ADF) method (Jones and Waller, 2015). Model evaluation included an examination of the model fit indices and squared multiple correlations. As widely recommended (Byrne, 2010; Mueller and Hancock, 2010; Garson, 2012; West et al., 2012) the goodness-of-fit of the factor structure was evaluated from three different perspectives (incremental, parsimonious, and absolute) using the normed chi-square value, the comparative fit index (CFI), the non-normed fit index (NNFI), the root mean square error of approximation (RMSEA), and the standardized root mean square residual (SRMR). Moreover, the measurement invariance across gender and work characteristics groups was analyzed through a multi-group CFA. After configural invariance across groups was established, testing for metric invariance and scalar invariance was performed using Δχ^2^ and ΔCFI tests for two nested models (Brown, 2006; Byrne, 2010; Dimitrov, 2010). A change of equal to or < 0.01 for CFI indicates that invariance holds (Cheung and Rensvold, 2002).

The internal consistency, concurrent and convergent validity of the scale were examined by Cronbach’s alpha coefficients and Spearman correlation coefficients, respectively. Moreover, correlational analyses and analysis of variance were used to perform group comparison.

## Results

### Item Analysis

Regarding the distributional properties of the 4 items, means ranged from 1.96 to 1.43 (see **Table 1**). When studying negative acts at work, such as WI, low means are common (e.g., Kabat-Farr et al., 2016). Standard deviations ranged from 0.99 to 0.78, skewness ranged from 0.90 to 2.45 and kurtosis ranged from 0.35 to 6.35. Some items are below indices for acceptable limits of 2 (Field, 2000, 2009; Trochim and Donnelly, 2006; Gravetter and Wallnau, 2014). Hence, as shown in **Table 1**, there is evidence of deviation of normal distribution.

**Table 1:** Means, standard deviation, item-adjusted total correlation, alpha if item deleted, and inter-item correlation of the workplace incivility scale.

Item	Mean	*SD*	Skewness *SE* = 0.121	Kurtosis *SE* = 0.241	*rit*	*αid*	Inter-item correlations		
							Item 1	Item 2	Item 3
Item 1	1.91	0.95	0.903	0.355	0.75	0.72			
Item 2	1.96	0.99	1.032	0.858	0.82	0.66	0.48		
Item 3	1.39	0.79	2.450	6.354	0.76	0.69	0.42	0.51	
Item 4	1.43	0.78	2.062	4.488	0.71	0.72	0.34	0.46	0.45
PL	2.45	1.2	0.456	-0.817					
JS	3.47	0.78	-0.463	-0.032					
PWB	2.99	0.49	-0.192	0.018					
SU	1.21	0.60	3.97	19.85					

*PL = Propension to leave; JS = Job Satisfaction; PWB = Psychological Well-being; SU = Social Undermining; *SD* standard deviation, *rit* item homogeneity*, αid* Alpha if-item-deleted. All correlations were statistically significant *p* < 0.01*.

### Confirmatory Factor Analysis

**FIGURE 1:**
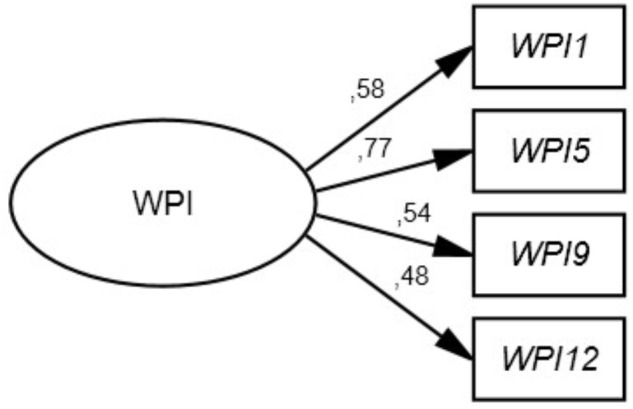
Standardized path loadings for 4-item scale. WI, Workplace Incivility.

Confirmatory factor analysis model with the unconstrained factor loadings are shown in **Figure 1**. The factor loadings ranged from 0.48 to 0.77 and parameters show a good fit (see **Figure 1**). Two CFAs were conducted for each pair of work characteristics –men vs. women; permanent vs. temporal contract; full-time vs. part-time and supervisor vs. non-supervisors- separately. Taken together, values suggest a good fit. A non-significant chi-square indicates a perfect fit, and a good fit is indicated when the ratio between the chi-square statistic and the degree of freedom is less than 5 (West et al., 2012). All CFI and NNFI values are > 0.95 indicating a good fit (Byrne, 2010). The RMSEA for the scale with all participants indicates a well-fitting model (Browne and Cudeck, 1993). With only one exception (for part-time employees), RMSEA values are lower 0.08, indicating an acceptable fitting (Hu and Bentler, 1999). All SRMR values are under 0.08 indicating a good fit (Hu and Bentler, 1999). Standardized residuals revealed that all values fell well below the 0.15, which is consistent with the notion of an acceptable fit (Byrne, 2010) (see **Figure 1**).

Based on selective discrimination’s assumptions, different ANOVA was conducted with all demographic variables: sex, age, studies, civil status, type of contract, employment status, responsibility position, and sector. There were no differences in WI perceptions.

Using the criterion by Nunnally (1978), the alpha coefficient of the reduced measure showed a good internal consistency (Cronbach’s α = 0.75). The mean and standard deviation of each item and the inter-item correlations are presented in **Table 1**. Sequential deletion of items generated alphas ranging from 0.66 to 0.72.

To test the convergent validity, Spearman’s correlation coefficients were computed between the reduced WIS and social undermining, the propensity to leave a job, job satisfaction and psychological well-being measures. In addition, guidelines Cohen’s (1988) were used to determine the strengths of the correlation coefficients between the reduced WIS and other measures, with *r* around 0.1 indicating small effect sizes, around 0.3 indicating medium effect sizes, and *r* > 0.5 indicating large effect sizes. Assessing for convergent validity, as expected, the reduced WIS had a large positive effect with social undermining. Regarding the concurrent validity, results showed that the scale was significantly, although moderately, associated with propensity to leave a job (*r* = 0.21, *p* < 0.01), job satisfaction (*r* = -0.44, *p* < 0.01) and psychological well-being (*r* = -0.20, *p* < 0.01) (see **Table 2**).

**Table 2:** Means, Standard Deviations, and Spearman Inter-correlations among variables (*N* = 407 participants).

Variable	alpha	Items	1	2	3	4
(1) Workplace Incivility	0.75	4				
(2) Social Undermining	0.96	26	0.50^∗∗^			
(3) Propensity to Leave a job	0.88	3	0.21^∗∗^	0.21^∗∗^		
(4) Job Satisfaction	0.77	5	-0.44^∗∗^	-0.30^∗∗^	-0.26^∗∗^	
(5) Psychological Well-Being	0.73	12	-0.20^∗∗^	-0.18^∗∗^	-0.05	0.37^∗∗^

^∗∗^*p < 0.01*.

To test measurement invariance across groups, configural, metric and scalar models were compared (see **Table 3**). The results showed that these four types of invariant models had a good fit in three out of four pair of groups analyzed (permanent vs. temporal, full-time vs. part-time and supervisor vs. non-supervisors groups). Multigroup analysis showed no invariance between men vs. women groups (see **Table 3**).

**Table 3:** Fit statistics for different work conditions employees.

Model	*N*	χ^2^	*df*	*p*	CFI	NFI	RMSEA	SRMR	COMPARISON	ΔCFI	Δχ^2^
Original model	407	1.420	2	0.492	1.000	0.980	0.000	0.012			
Permanent Contract	294	2.470	2	0.291	0.990	0.953	0.028	0.022			
Temporal Contract	77	1.843	2	0.398	1.000	0.910	0.000	0.034			
Full-time	286	2.210	2	0.330	0.996	0.960	0.020	0.021			
Part-time	121	3.730	2	0.154	0.928	0.877	0.085	0.072			
Supervisors	171	0.775	2	0.685	1.000	0.969	0.000	0.017			
Non-supervisors	234	2.005	2	0.367	1.000	0.962	0.020	0.003			
Women	241	7.643	2	0.220	0.876	0.851	0.108	0.033			
Men	166	3.294	2	0.193	0.966	0.925	0.063	0.037			
Invariance model											
Model 1a		3.829	4	0.147	0.970	0.948	0.050	0.029	2 versus 1	0.030	χ^2^ (4) = 1.778, *p* > 0.05
Model 2a		5.607	8	0.469	1.000	924	0.000	0.055	3 versus 1	-0.065	χ^2^ (8) = 13.019, *p* > 0.05
Model 3a		16.848	12	0.112	0.905	0.770	0.038	0.077	3 versus 2	-0.095	χ^2^ (4) = 11.241, *p* > 0.05
											
Model 1b		4.06	4	0.397	0.999	0.946	0.007	0.028	2 versus 1	0.001	χ^2^(4) = 1.85, *p* > 0.05
Model 2b		5.919	8	0.656	1.000	0.921	0.000	0.050	3 versus 1	0.001	χ^2^(8) = 7.36, *p* > 0.05
Model 3b		11.429	12	0.487	1.000	0.847	0.000	0.054	3 versus 2	0.000	χ^2^ (4) = 5.51, *p* > 0.05
											
Model 1c		2.760	4	0.599	1.000	0.964	0.000	0.018	2 versus 1	0.000	χ^2^(4) = 4.70, *p* > 0.05
Model 2c		7.460	8	0.488	1.000	0.904	0.000	0.060	3 versus 1	0.000	χ^2^(8) = 6.99, *p* > 0.05
Model 3c		9.759	12	0.637	1.000	0.874	0.000	0.065	3 versus 2	0.000	χ^2^(4) = 2.29, *p* > 0.05

*a = Permanent contract versus temporal contract; b = full-time versus part-time employees; c = supervisors versus non-supervisors. Model 1 = Configural model; Model 2 = Metric model; Model 3 = Scalar model*.

In the original model, CFA for men showed a good fit (see **Table 3** for the fit indices). However, the CFA for the original model did not fit for women (see **Table 3**). Therefore, no invariance models between men and women are analyzed. According with Byrne (2010), we can conclude about metric invariance for permanent vs. temporal contract groups. Although the value of the ΔCFI was below the cut-off value (ΔCFI = 0.030), Δχ^2^ is not significant (1.77, *p* > 0.005), showing invariance. For full-time vs. part-time and supervisor vs. non-supervisors groups, when configural and metric invariant models were compared, no significant changes occurred in fit indices. Both ΔCFI and Δχ^2^ were smaller than the cut-off values (ΔCFI = 0.001 and Δχ^2^ = 1.85, *p* > 0.05, for full-time vs. part-time groups; and ΔCFI = 0.000 and Δχ^2^ = 4.70, *p* > 0.05, for supervisor vs. non-supervisors groups). These results suggest that factor loadings were invariant across the each pair of groups.

When metric and scalar invariant models were compared, neither significant changes occurred in fit indices (ΔCFI = 0.000 and Δχ^2^ = 5.51, *p* > 0.05, for full-time vs. part-time groups; and ΔCFI = 0.000 and Δχ^2^ = 2.29, *p* > 0.05, for supervisor vs. non-supervisors groups). These results suggest that factor loadings and intercepts were invariant across the each pair of groups. However, for permanent vs. temporal contract, again, although Δχ^2^ = 11.241, *p* > 0.05, ΔCFI = -0.095, so invariance could not be concluded.

## Discussion

The goal of this article was to validate the reduced version of the WIS from English to Spanish. After a rigorous process of translation, the validity of the scale was statistically demonstrated. Similarly to the English version (Matthews and Ritter, 2016), the scale shows a good reliability. The convergent and the concurrent validity of the scale was explored by analyzing its relation with other constructs such as social undermining, the propensity to leave a job, the job satisfaction and the psychological well-being. As expected, the reduced WIS was positively associated with the first variable and negatively associated with the last two ones. Thus, although the participants’ mean scores on WI are not high (*M* = 1.67), WI produces negative consequences for people and organizations. Low participants’ mean scores are in line with previous research (Diaz et al., 2011; Garrosa et al., 2015; Kabat-Farr et al., 2016) and might be due to the low intensity and ambiguity that characterize uncivil behaviors and which make incivility less recognizable.

The unidimensional model tested showed a minimum factor loading higher than the one of the original scale (0.61 vs. 0.57). The goodness-of-fit of the factor structure was good.

The contribution of this work goes beyond testing the validity of WIS in Spain. In fact, our results also show the invariance of the Spanish reduced scale throughout workers with different employment status. Specifically, two CFAs were carried out for each work characteristics respectively, demonstrating the invariance of the scale between permanent versus temporal, full-time versus part-time, and supervisor versus non-supervisor workers. Subordinates, people with a part-time or temporal contract might be victims of negative and uncivil acts more frequently; moreover, the types of uncivil behaviors that they can experience might be different than those ones experiences by colleagues in other contract/hierarchical position. This scale overcomes limitations derived by the employment status, offering a valid invariant tool for people at any level of the hierarchical position and with different contract type or working hours per week. The scale was not gender invariant demonstrating that men and women experience WI differently. Future research should increase the sample size in order to test again gender invariance.

Another advantage of this scale is its length. This short version increases the probability to obtain a high response rate when several variables are measured at the same time (Galiana et al., 2015), or a longitudinal study is carried out. Moreover, the low number of items makes it easy to answer by computer. Furthermore, its psychometric properties have been tested with workers of different sectors, which guarantee its functionality and validity beyond the context where the scale is used.

This research has several theoretical and practical implications. At a theoretical level, it allows advancing in the field of WI, offering a scale for studies carried out with Spanish speaking workers and providing an invariant measure across the employment status.

Although the Spanish reduced version of the WIS is a starting point for researchers who are interested on uncivil acts, we have to acknowledge that this instrument does not allow exploring other aspects of this phenomenon, accordingly with the new nature of the work. In fact, recent research (Park et al., 2015; Krishnan, 2016) has underlined modern forms of incivility, connected with new technologies. Cyber incivility (such as sending rude email or using all caps in order to transmit a reproach) is widespread and its consequences are still largely unknown. The next step in this direction should be the study of psychometric properties of cyber incivility instruments that already exist.

At a practical level, this research provides to Human Resources Managers an easy tool for the evaluation of uncivil behaviors at the workplace, in order to take measures when a climate of incivility is perceived. In fact, perceiving uncivil behaviors at work reduces employees’ well-being and job satisfaction (Miner and Cortina, 2016) which, in turn, affect employees’ quality of life. Moreover, the outcomes of negative work dynamics might spill onto the personal domain, deteriorating people quality of life (De Simone et al., 2014).

A limitation of this study might be the unique recall window that has been analyzed. While previous research (Matthews and Ritter, 2016), tested the factor structure of the reduced scale using three different recall windows (2 weeks, 1 month, and 1 year), in this study participants were asked about their experience of incivility in the ‘last year’. However, Matthews and Ritter (2016) showed that there exist no differences in the factor structure of the reduced scale with those three different recall windows. Notwithstanding, it is necessary to take into account that “one month” recall window might be the best time frame to provide a realistic experience of uncivil acts (Matthews and Ritter, 2016).

To sum up, this study validates the reduced version of WIS in a sample of workers from several sectors in Spain. The measure embraces four different behaviors that are considered uncivil invariably by people in different employment status. As expected the reduced Spanish measure correlates negatively with job satisfaction and well-being and positively with propensity to leave.

## Ethics Statement

The Ethics Committee of the Faculty of Psychology (University of Seville, Spain) approved the study.

## Author Contributions

All the authors contributed to the conception and design of the work and the acquisition, analysis, and interpretation of data for the work. They drafted the work and revised it critically. The authors gave the final approval of the manuscript before the submission. They participated at any step of the research.

## Conflict of Interest Statement

The authors declare that the research was conducted in the absence of any commercial or financial relationships that could be construed as a potential conflict of interest.

## Appendix

Spanish and English items of the reduced version of the workplace incivility scale.

(1) ¿Ha/n prestado poca atención a sus ideas o ha/n mostrado poco interés en sus opiniones?
     *Paid little attention to your statements or showed little interest in your opinions.*
(2) ¿Le ha/n interrumpido o ha/n comenzado a hablar mientras usted hablaba?
     *Interrupted or “spoke over” you.*
(3) ¿Le ha/n ignorado o ha/n dejado de hablarle (por ejemplo: le ha/n hecho el vacío)?
     *Ignored you or failed to speak to you (e.g., gave you “the silent treatment”).*
(4) ¿Han/n hecho bromas a su costa?
     *Make jokes at your expense.*
